# Prophylactic intra-aortic balloon counterpulsation in cardiac surgery: it is time for clear evidence

**DOI:** 10.1186/s13054-014-0662-2

**Published:** 2014-11-29

**Authors:** Philippe Grieshaber, Bernd Niemann, Peter Roth, Andreas Böning

**Affiliations:** Department of Adult and Pediatric Cardiovascular Surgery, University Hospital Giessen, Rudolf-Buchheim-Strasse 8, Giessen, DE-35392 Germany

## Abstract

In the previous issue of *Critical Care*, Yu and colleagues report increased morbidity and mortality in patients after myocardial infarction undergoing prophylactic intra-aortic balloon pump support before coronary artery bypass graft surgery. The impact of prophylactic intra-aortic balloon pump implantation before coronary artery bypass graft therapy still is controversially debated. However, Yu and colleagues emphasize further discussion and substantiate the need for a prospective randomized controlled trial on this subject.

Yu and colleagues investigate in their well-conducted retrospective analysis the impact of preoperative insertion of an intra-aortic balloon pump (IABP) on postoperative outcome in patients undergoing coronary artery bypass graft (CABG) after acute myocardial infarction [1]. The rationale for prophylactic IABP is to decrease the left ventricular afterload and to improve coronary perfusion, and thus to reduce the risk of perioperative low cardiac output syndrome. In this study, preoperative IABP was associated with increased morbidity, transfusion requirements and longer stay on the ICU postoperatively.

The use of prophylactic IABP in patients undergoing CABG is based on data generated by Christenson and colleagues in the late 1990s and early 2000s showing positive effects of prophylactic IABP insertion on postoperative short-term and long-term survival [2-5]. However, these positive results have been challenged in the past few years by a number of retrospective analyses showing conflicting results [6-9]. A recent prospective randomized study by Ranucci and colleagues showed no positive effect of prophylactic IABP in patients with severely reduced left ventricular function undergoing CABG [10]. However, this study had several limitations we have addressed elsewhere [11].

The current study by Yu and colleagues revealed an association between preoperative IABP and a prolonged stay on the ICU. This result is not surprising, as postoperative weaning from an IABP, which usually takes 24 to 48 hours, has to be performed under intensive care monitoring. The composite morbidity endpoint was reached more often in the IABP group. The authors also observed increased transfusion requirements in the IABP group that might, as the authors state, be related to mechanical thrombocyte consumption and hemolysis rather than bleeding complications from IABP insertion. Increased transfusion of erythrocytes has been associated with poor short-term and long-term outcome after cardiac surgery in previous studies [12,13]. Interestingly, neither increased transfusion nor the other parameters investigated in this study influenced the overall length of hospital stay and in-hospital mortality. Contrarily, the excellent short-term mortality rates in both groups (control group, 1.0%; IABP group, 2.5%) suggest that this study population did not represent a true high-risk patient collective.

On the one hand, Yu and colleagues’ study adds some interesting results; on the other, numerous issues about prophylactic IABP use still remain unanswered. The current uncertainty about prophylactic IABP use in patients undergoing CABG results from different aspects. In all studies so far conducted, the criteria for prophylactic IABP insertion were not well defined and were based on subjective decisions by the treating physicians. Accordingly, the term high-risk patient was based on very individual criteria rather than on established tools for perioperative risk estimation (for example, EuroSCORE, STS Risk Score). The optimal timing of IABP insertion still is not known, although some authors showed benefit from early insertion [3,9]. However, the ideal length of temporization of patients with myocardial infarction before CABG is also subject to individual perception rather than to clinical evidence [14-16]. Finally, the value of prophylactic IABP has to be judged in the context of the best-valued alternative therapy.

From a pathophysiological point of view, we suppose that the effects of IABP (increased coronary perfusion, reduction of left ventricular afterload) might induce the greatest effect in patients with critically reduced coronary perfusion and (temporarily) severely reduced left ventricular function; that is, in patients with acute myocardial infarction who need to be temporized prior to CABG. In some institutions, including our clinic, prophylactic IABP in these patients is initiated in the catheter laboratory or, at the latest, after admission to the ICU. The patients, if hemodynamically stable and without ongoing symptoms, are then temporized until cardiac enzymes are recurrent, which usually takes 2 to 3 days. However, regarding the paucity of good and contemporary data on the value of prophylactic IABP, this clinical routine lacks evidence.

The best way to obtain evidence and to generate reliable guidelines for the prophylactic use of IABP in patients undergoing CABG is an adequately powered prospective randomized controlled trial with a well-defined study population, with a standardized protocol for perioperative care and IABP handling and with a clinically relevant and appropriate primary endpoint (for example, 30-day mortality).

## Abbreviations

CABG: Coronary artery bypass graft
IABP: Intra-aortic balloon pump

## References

[1] Yu P-J, Cassiere HA, Dellis SL, Kohn N, Manetta F, Hartman AR (2014). Propensity-matched analysis of the effect of preoperative intraaortic balloon pump in coronary artery bypass grafting after recent acute myocardial infarction on postoperative outcomes. Crit Care.

[2] Christenson JT, Badel P, Simonet F, Schmuziger M (1997). Preoperative intraaortic balloon pump enhances cardiac performance and improves the outcome of redo CABG. Ann Thorac Surg.

[3] Christenson JT, Simonet F, Badel P, Schmuziger M (1999). Optimal timing of preoperative intraaortic balloon pump support in high-risk coronary patients. Ann Thorac Surg.

[4] Christenson JT, Cohen M, Ferguson JJ, Freedman RJ, Miller MF, Ohman EM, Reddy RC, Stone GW, Urban PM (2002). Trends in intraaortic balloon counterpulsation complications and outcomes in cardiac surgery. Ann Thorac Surg.

[5] Christenson JT, Licker M, Kalangos A (2003). The role of intra-aortic counterpulsation in high-risk OPCAB surgery. J Card Surg.

[6] Miceli A, Fiorani B, Danesi TH, Melina G, Sinatra R (2009). Prophylactic intra-aortic balloon pump in high-risk patients undergoing coronary artery bypass grafting: a propensity score analysis. Interact Cardiovasc Thorac Surg.

[7] Qiu Z, Chen X, Xu M, Jiang Y, Xiao L, Liu L, Wang L (2009). Evaluation of preoperative intra-aortic balloon pump in coronary patients with severe left ventricular dysfunction undergoing OPCAB surgery: early and mid-term outcomes. J Cardiothorac Surg.

[8] Santarpino G, Onorati F, Rubino AS, Abdalla K, Caroleo S, Santangelo E, Renzulli A (2009). Preoperative intraaortic balloon pumping improves outcomes for high-risk patients in routine coronary artery bypass graft surgery. Ann Thorac Surg.

[9] Böning A, Buschbeck S, Roth P, Scheibelhut C, Bödeker RH, Brück M, Niemann B (2013). IABP before cardiac surgery: clinical benefit compared to intraoperative implantation. Perfusion.

[10] Ranucci M, Castelvecchio S, Biondi A, de Vincentiis C, Ballotta A, Varrica A, Frigiola A, Menicanti L, Surgical and Clinical Outcome Research (SCORE) Group (2013). A randomized controlled trial of preoperative intra-aortic balloon pump in coronary patients with poor left ventricular function undergoing coronary artery bypass surgery. Crit Care Med.

[11] Grieshaber P, Böning A (2014). Prophylactic intra-aortic balloon counterpulsation in cardiac surgery: challenges in planning the ‘right’ trial. Crit Care Med.

[12] Koch CG, Li L, Duncan AI, Mihaljevic T, Loop FD, Starr NJ, Blackstone EH (2006). Transfusion in coronary artery bypass grafting is associated with reduced long-term survival. Ann Thorac Surg.

[13] Loor G, Li L, Sabik JF, Rajeswaran J, Blackstone EH, Koch CG (2012). Nadir hematocrit during cardiopulmonary bypass: end-organ dysfunction and mortality. J Thorac Cardiovasc Surg.

[14] Lee DC, Oz MC, Weinberg AD, Ting W (2003). Appropriate timing of surgical intervention after transmural acute myocardial infarction. J Thorac Cardiovasc Surg.

[15] Caceres M, Weiman DS (2013). Optimal timing of coronary artery bypass grafting in acute myocardial infarction. Ann Thorac Surg.

[16] Weiss ES, Chang DD, Joyce DL, Nwakanma LU, Yuh DD (2008). Optimal timing of coronary artery bypass after acute myocardial infarction: a review of California discharge data. J Thorac Cardiovasc Surg.

